# Community health worker interventions to promote psychosocial outcomes among people living with HIV—A systematic review

**DOI:** 10.1371/journal.pone.0194928

**Published:** 2018-04-24

**Authors:** Hae-Ra Han, Kyounghae Kim, Jeanne Murphy, Joycelyn Cudjoe, Patty Wilson, Phyllis Sharps, Jason E. Farley

**Affiliations:** 1 The Johns Hopkins University, School of Nursing, Baltimore, Maryland, United States of America; 2 Center for Cardiovascular and Chronic Care, The Johns Hopkins University, Baltimore, Maryland, United States of America; 3 University of Connecticut, School of Nursing, Storrs, Connecticut, United States of America; 4 The George Washington University School of Nursing, Ashburn, Virginia, United States of America; 5 The REACH Initiative, The Johns Hopkins University, Baltimore, Maryland, United States of America; TNO, NETHERLANDS

## Abstract

**Background:**

Community health worker (CHW) interventions are a successful strategy to promote health among HIV-negative and persons living with HIV (PLWH). Psychosocial factors are critical dimensions of HIV/AIDS care contributing to prognosis of the disease, yet it is unclear how CHW interventions improve psychosocial outcomes in PLWH. The purpose of this study was to critically appraise the types, scope, and nature of CHW interventions designed to address psychosocial outcomes in PLWH.

**Methods:**

We performed database searches—PubMed, EMBASE, CINAHL, and Cochrane—to identify randomized controlled trials published in English before April 2017. Fourteen articles met the eligibility criteria.

**Results:**

Half of the studies were conducted in the United States. Social cognitive theory was used more than once in nine theory-guided studies. CHW interventions were largely focused on reducing depression (n = 6) or stigma related to HIV (n = 4), or promoting quality of life (n = 4), social support (n = 4), and self-efficacy (n = 4). Didactic methods and role-playing were used to train CHWs. CHWs played multiple roles in delivering intervention, including a counselor and a supporter (n = 10), educator (n = 5), or a navigator (n = 3). CHW intervention fidelity was assessed in 4 studies. Five studies found positive changes in six psychosocial outcomes including quality of life (2 of 4) and self-efficacy (2 of 4). CHW interventions had no effect on social support in 2 of 4 studies, and stigma in 3 of 4 studies. None of the CHW interventions were successful in reducing depressive symptoms among PLWH.

**Conclusions:**

Evidence partially supported the use of CHWs in promoting psychosocial outcomes in PLWH. Future CHW intervention should be expanded in scope to address key psychosocial determinants of HIV/AIDS outcomes such as health literacy. Further, fidelity measures should be incorporated into intervention delivery.

## Background

Community health workers (CHWs) are individuals who share the same ethnicity, language, culture, sexual orientation or geographic community of the patients they serve. Hence, they are uniquely aware of the ethnic, linguistic, socioeconomic, cultural, and experiential factors that may influence that community’s use of healthcare services [1]. With their unique ability to bridge the community and healthcare services, CHWs convey health information and resources in diverse settings. CHW interventions have been employed as a successful strategy to address physical and psychosocial needs of individuals with chronic diseases including HIV in resource-poor communities. Specifically, in addition to participant recruitment and data collection, CHWs assume a wide range of roles including counseling, education, navigation assistance, case management, HIV testing, adherence support for antiretroviral therapy, and social services including vocational training for people living with HIV (PLWH) [2, 3]. Overall, CHW interventions tend to be effective in cardiovascular risk reduction and cancer prevention [2, 4], reduction of depression [5], and among PLWH, enhancing the uptake (e.g., HIV testing, picking up rates of antiretroviral drugs, self-reported adherence rates to antiretroviral therapy), and quality (reduced waiting times, improved patient flow, improvement in the filling of medical records) of HIV services [3, 6].

The World Health Organization underscores the importance of psychosocial support for PLWH and recommends psychosocial support by CHWs as part of guidelines for the management of HIV/AIDS [7]. This is because psychosocial factors are critical dimensions of HIV/AIDS care contributing to prognosis of the disease. For example, Bing and colleagues [8] assessed a national probability sample of nearly 3,000 PLWH and found that more than a third screened positive for clinical depression, the most common disorder identified. PLWH often confront a range of psychological challenges, the fear of physical decline and worsening quality of life, treatment burden, and coping with uncertainty [8]. Further, HIV infection and/or AIDS is often associated with both perceived and enacted stigma which fosters challenges of adherence through the treatment cascade [9]. Achieving viral suppression by maintaining adherence to lifelong therapy may be complicated by each of these dimensions. In particular, addressing psychosocial needs through proper support can help PLWH and their partners and caregivers cope more adequately with each stage of HIV infection, promote quality of life, and respond properly to the stress related to HIV infection [7]. This highlights the need for a comprehensive investigation of psychosical factors that are targeted by CHW interventions, their effects on psychosocial outcomes, and the specific roles of CHWs, thereby informing further CHW research to more effectively address psychosocial needs of PLWH.

Previous systematic reviews have revealed that CHW interventions are effective in HIV prevention [10, 11]; linkage to HIV care [3, 6, 12]; and medication adherence among patients with HIV and AIDS [3, 6, 13]. The World Health Organizations calls for improvement in the training and education of CHWs so they can provide quality care to vulnerable and hard-to-reach communities [6]. Despite the psychosocial challenges confronted by PLWH and the emphasis on psychosocial support by CHWs for the management of HIV/AIDS [7], none of these reviews specifically targeted psychosocial outcomes of CHW interventions, nor did they investigate the training of CHWs. CHWs are ideally positioned to address psychosocial issues due to their understanding of the complex health and social needs of PLWH as indigenous, frontline health personnel [1, 2]. This unique set of strengths may yield a beneficial effect on psychosocial outcomes. To this end, the purpose of this study was to critically appraise the types, scope, and nature of CHW interventions designed to address psychosocial outcomes in PLWH. The term, psychosocial factors, has been variedly used within health care research [14]. For the purpose of our review, ‘psychosocial’ was used as an umbrella term pertaining to ‘the interrelation of social factors and individual thought (e.g., perception, belief, attitude) and behaviour’ relative to HIV/AIDS prevention and care [15] with examples of psychosocial variables including: stigma, depression, self-efficacy, adherence, or quality of life. We aimed to explain the types of CHW interventions designed to address psychosocial outcomes in PLWH; describe training and roles of selected CHWs; and evaluate the psychosocial outcomes of CHW interventions among PLWH. This systematic review extends previous reviews by providing a comprehensive understanding of: 1) how CHWs are trained prior to the delivery of an intervention; 2) how CHWs implement an assigned intervention; and 3) how CHWs interventions achieve desired psychosocial effects.

## Methods

Electronic databases—PubMed, EMBASE, CINAHL, and Cochrane, were searched to identify randomized controlled trials published in English from inception (1988 for PubMed, 1987 for EMBASE, and 1952 for CINAHL) to December 2015. Thirteen articles met the eligibility criteria. The searches were updated to identify additional randomized controlled trials published between January 2016 and March 2017. One additional article was added, resulting in a total of 14 articles.

### Review design

We undertook a systematic review of quantitative evidence of randomized clinical trials (RCTs) that are designed to change psychosocial outcomes for PLWH. Due to heterogeneity relative to psychosocial outcomes and statistical analyses approaches among the included studies, we qualitatively synthesized the study findings rather than conducting meta-analysis.

### Study eligibility

#### Inclusion criteria

Studies were screened to assess their relevance to the purpose of our review. Specifically, the following inclusion criteria were used: (1) articles that use a randomized controlled trial (RCT); (2) articles testing CHW-led interventions on noncommunicable chronic conditions; and (3) articles including adult study participants with HIV and AIDS. Only RCTs were included in order to build the strongest evidence to rigorously evaluate the causality between CHW interventions and psychosocial outcomes. In addition, our search did not specify the types of chronic conditions in order to avoid missing any relevant articles. To maximize the breadth of the study findings, we included any study that reported quantitative findings relevant to the review question. Studies from around the globe were included, as were studies conducted in various settings including community or health system settings. Only studies written in English were included.

#### Exclusion criteria

Studies were excluded if they focused on PLWH but did not address any psychosocial variables. In addition, we excluded studies if: full-texts were unavailable (i.e., conference abstracts), they were not randomized controlled trials, or studies which reported protocol only with no reported outcomes.

### Search strategy and selection process

#### Search strategy

In consultation with a medical librarian, peer-reviewed journal articles were searched systematically in the PubMed, CINAHL, Embase, and Cochrane databases using variations of MeSH terms: population of interest (i.e., PLWH); study design of interest (i.e., RCT); and intervention method of interest (i.e., CHW). A full search strategy for the database searches can be found in Appendix 1. Articles and abstracts were excluded if they did not address the population, interventions or methodology of interest.

#### Study selection

**Fig 1:**
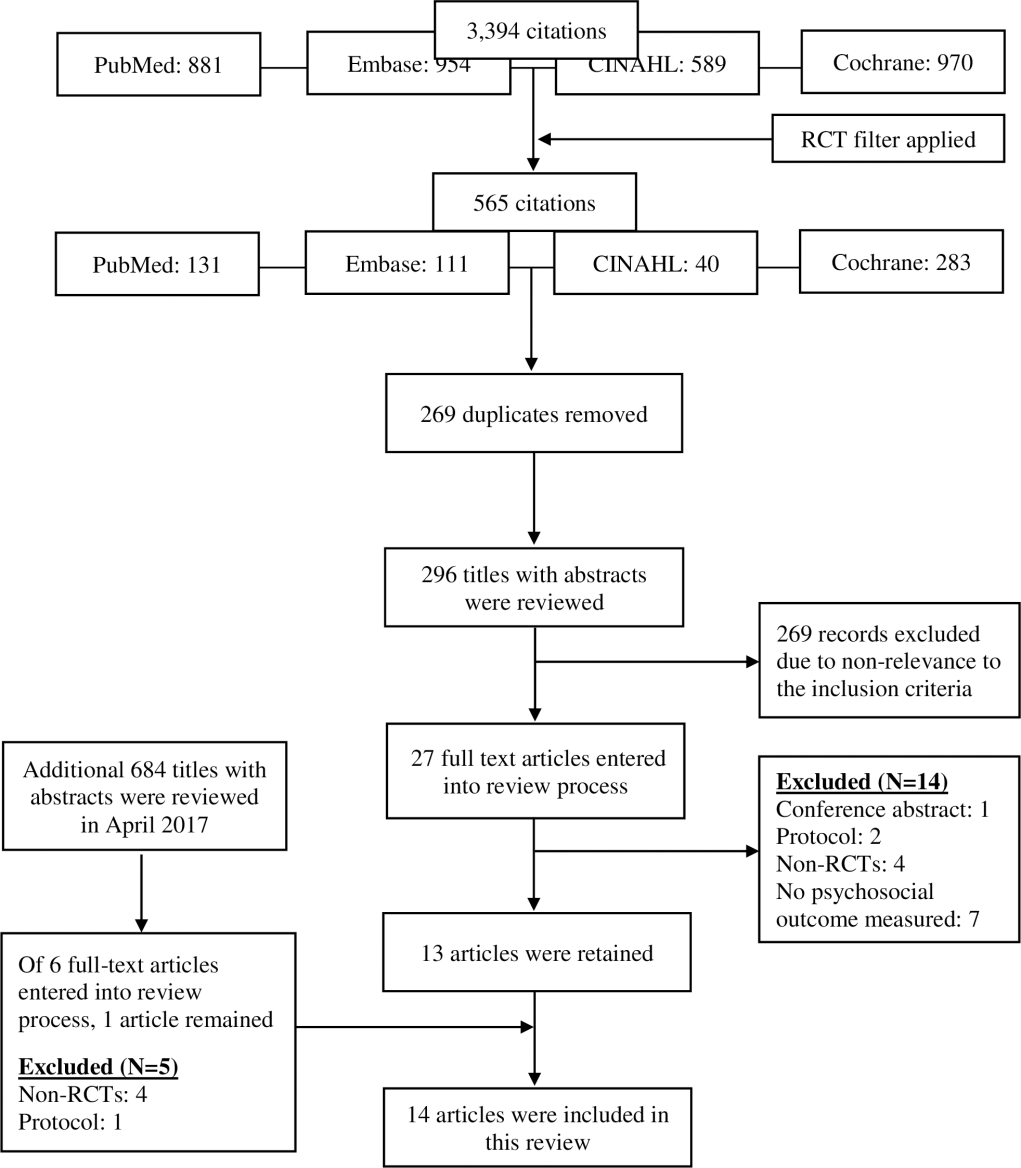
Review and selection process.

A detailed outline of the article selection process is offered in Fig 1. Our initial database search in December 2015 resulted in a total of 3,394 citations. After applying a filter for limiting searches to RCT, 565 citations remained. After removing duplicates, 296 titles with abstracts were reviewed independently by two authors for relevance. A total of 27 articles were passed onto the next full text review process. Of 27 full-text articles that were reviewed thirteen were deemed eligible. Reasons for exclusion included no psychosocial outcome measured (n = 7), non-RCTs (n = 4), protocol (n = 2), and conference abstract (n = 1). Using the same search terms (Appendix 1), an additional database search was conducted in April 2017 for the studies published since January 2016. After removing duplicates, 684 titles with abstracts were reviewed for relevance. A second independent level of review was performed on 6 full text review of 6 articles that met the inclusison criteria. As a result of additional searches, one article was additionally included after eliminating 5 full-text articles that were entered into review process. Reasons for exclusion from the expanded search were: non-RCTs (n = 4) and protocol (n = 1).

### Quality assessment and data extraction

#### Characterizing the evidence base

**Fig 2:**
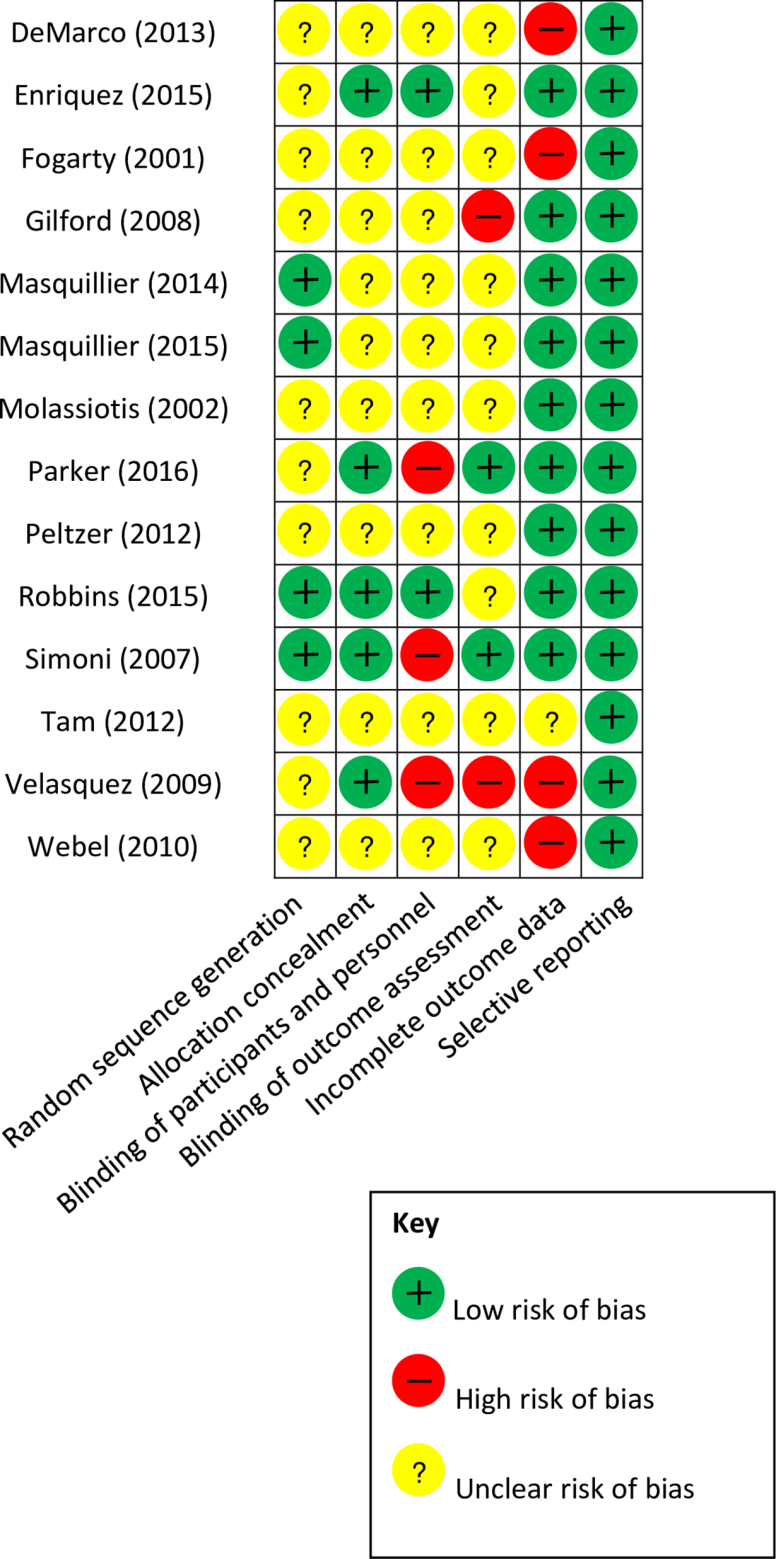
Methodological quality ratings of included studies.

In order to assess the rigor of the underlying evidence base for the review, we first developed an overview of key methodolotical characteristics of the included studies by summarizing several variables, including study design, sample size and strategy, study setting, and risk of bias. We then ranked each included study as low, high, or unclear with respect to overall risk of bias using the Cochrane Collaboration’s tool for assessing risk of bias [16]. This tool assesses for risk of selection bias, performance bias, detection bias, attrition bias, and reporting bias. The presentation of assessments of risk of bias can be an illustration (i.e., a pictograph) and/or a table including judgments (low risks, high risks, or unclear risks) with support for judgment. Quality of each study was rated independently by two raters with a 92.3% agreement rate (Kappa 0.77, p<0.001). Kappa statistics calculated using Stata 14. Two raters discussed and then resolved discrepancies. Given the widely varying study types, these rankings were based on criteria appropriate to each study design. The rankings were further justified via short narratives. This provided an overview of the quality of the existing evidence base, as represented by the included studies. No studies were excluded on the basis of the quality assessment. Rather, the quality assessment process was used to identify strengths and weaknesses in study methodologies and to guide the interpretation and assessment of study findings. The results of risk of bias assessments are shown in Fig 2. Overall, the methodological rigor of the included studies was poor to moderate. No study had low risk of bias in all cateogories, and seven studies had a high risk for bias in at least one category [17–23]. Most studies did not provide sufficient information to allow raters to determine bias in their randomization and blindness of study participants. In addition, three studies [17, 22, 24] used per-protocol analyses. All primary and secondary outcomes were reported in the included studies regardless of its significance. Six studies conducted a priori power analysis [17, 20–23, 25].

#### Data extraction and management

Once the study selection process was concluded, one review author extracted data from the studies using a standard template. Initial data extraction captured both the study characteristics (e.g., setting, participants, and type of intervention reviewed) as well as key findings related to the intervention. Other team members also reviewed the studies and extracted data relating to key findings and relevant variables associated with the intervention. Extracted findings were compared, and discussed until all discrepancies resolved.

## Results

### Overview of studies included and CHW interventions

Table 1 summarizes the main characteristics of included studies. We included 13 unique articles [17–29] with one companion article [30]. One companion article [30] presented the secondary analysis of the main study [29]. Seven studies were conducted in the U.S.; five were in South Africa [20, 25, 26, 29, 30]; two were conducted in Asia including Vietnam [24] and China [27]. Four studies [17, 18, 20, 23] exclusively included female participants while two studies included male participants only [19, 22]. Sample sizes varied widely from 20 [28] to 498 [29]. The samples ranged in age from 30 years [18] to 47 years [23]. Study sites involved outpatient clinics [21, 23, 24, 28], public antiretroviral treatment (ART) clinics [26, 29, 30], hospital-based ART clinics [18, 25], community-based organizations [17, 20], participants’ homes [24], or homeless shelters [18]. Studies also described recruitng study particiapnts by using public service announcements, ads in community newspapers, and pamphlets distributed at HIV clinics [19]. Social Cognitive Theory was used in 3 of 9 theory-guided studies [19, 23, 28]. Transtheoretical model [18], Wellness Motivation Theory [28], Socio-ecological perspective and hope as a framework for HIV prevention and treatment [29], Health Belief Model and Health Promotion Model [25], Social Action Theory [26], and International Classification of Functioning, Disability and Health [20] were also used.

**Table 1:** Study characteristics.

1^st^ author (Year)^ref^	Setting/Sample	Intervention	Control	Psychosocial outcome(s)/ Instrument(s)	Main results
DeMarco (2013)[17]	Setting: Community-based women's drop-in center in an urban, black neighborhood of Boston, Massachusetts. Sample: Low-income, black women living with HIV infection (N = 110; Int: 56; Cont: 54). Mean age: 44.6y <HS graduate: 31.8%	Peer-led, small-group, structured writing using film clips from Women's Voices Women's Lives as a writing prompt	Attention control support	Self-advocacy: Silencing the Self Scale HIV stigma: HIV Stigma Scale	No between group difference in self-advocay and HIV stigma at 6-mo
Enriquez (2015)[28]	Adult patients linked to HIV medical care without suppressed viral load (N = 20). People of color: 75% About half of participants: >45 y	A peer-led HIV medication adherence intervention named 'Ready' or a time equivalent 'healthy eating' control arm. Lay individuals living with HIV were trained to facilitate 'Ready'.	Healthy eating control	Readiness for the healthy behavior change of adherence: Index of Readiness Social support: Medical Outcomes Study-Social Support survey Depression: CES-D	No between group differences in readiness, social support, and depressive symptoms at 6-mo
Fogarty (2001)[18]	Sample from two studies were used: HIV-infected women in one study (N = 124; black: 91%; mean age: 32y; <HS: 49%) and women at high risk for HIV infection in a second study (N = 843; black: 89%; mean age: 30y; <HS:56%)	An enhanced intervention included support groups and one-on-one contacts with peer advocates tailored to clients' needs.	Standard treatment	Stage of change (three behaviors: change in stage of behavior for condom use with a main male partner; condom use with other male partners; and contraceptive use) Self-efficacy for condom use Perceived advantages & disadvantages of condom and contraceptive use	Int group showed greater increases in self-efficacy for condom use between 6-mo and 12-mo (OR: 7.36, *p*<0.001) vs Cont
Gifford (1998)[19]	Setting: San Francisco Bay communities Sample: Symptomatic HIV or AIDS (N = 71) Int (n = 34): mean age: 45.2y; race: white: 68%; <HS graduate: 16% Cont (n = 37): mean age: 45.3y; race: white: 82%; <HS graduate: 9%	Interactive health education (7 sessions) to learn wide-ranging disease self-management skills and information: symptom assessment and management, medication use, physical exercise, relaxation, doctor-patient communication, and nutrition. Each group was led by two trained peer-leaders (one of whom was HIV-positive)	Usual care	Self-efficacy for controlling symptoms: Symptom self-efficacy items Depression: CES-D Stress: Perceived Stress Scale Anger: 8-item subscale drawn from the Chesney et al. Burn Out Scale	Int group showed greater increases in self-efficacy for controlling symptoms at 3-mo vs Cont (+4 vs. -7; *p*< .02) No between group difference in depression and psychosocial distress/stress
Masquillier (2014)[29]	Participants were recruited from 12 public antiretroviral treatment (ART) clinics across five districts in the Free State Province of South Africa (N = 498) Mean age: 38.9y; female: 77.4%; <HS graudate: 76.9%	Standard care + A group receiving additional biweekly peer adherence support (PAS) or a group receiving PAS and nutritional support.	Standard care	Hope: Adult State of Hope Scale	No between group difference in the level of hope at about 30-mo (2^nd^ follow-up)
Masquillier (2015)[30] (secondary analysis)	294 PLWHA from a randomized controlled trial; Participants (18+y) having commenced ART in the past 5 weeks were selected from 12 public ART clinics across five districts in the Free State Province of South Africa. Mean age: 38.97y; female: 75.1%; <HS graduate: 79.1%	Standard care + A group receiving additional biweekly peer adherence support (PAS) or a group receiving PAS and nutritional support.	Standard care	Treatment buddy (Informal social support) Internalized stigma (felt stigma): Berger’s HIV Stigma Scale	The intervention had a positive impact on seeking treatment buddying (β = 0.265, *p* = 0.007), which then leads to decreases in the levels of felt stigma (β = -0.149, *p* = 0.009 at about 30-mo (2^nd^ follow-up)
Molassiotis (2002)[27]	46 Chinese patients with symptomatic HIV Mean age: 39.1y; female: 8.6%; <HS graudate: 28.6%	Cognitive-behavioral group therapy (CBT): 12 weekly sessions of therapy over 3 months Peer support/ counseling group therapy	Comparison group	Psychological functioning: Profile of Mood States (anger, tension-anxiety, depression, confusion, overall mood) Quality of life: WHOQOL -BREF Uncertainty in illness: Mishel Uncertainty in Illness Scale	No between group difference in quality of life among three groups at 3-mo
Parker (2016)[20]	27 HIV-infected amaXhosa women aged 18–40 years in resource poor community in Cape Town, South Africa Mean age: 30.8y; numbers of years in school: 10.5y	Peer-led aerobic and strengthening exercise plus educational component on problem solving and goal setting (n = 12)	Educational component only (n = 15)	Self-efficacy, depression, quality of life	No between group difference in self-efficacy, depression, and quality of life at 4-mo
Peltzer (2012)[25]	152 adult patients on ART and with adherence problems at an HIV clinic in a district hospital in South Africa Mean age: 36.9y; female: 65.13%; <HS graduate: 50.66%	A standard adherence intervention package plus a structured three session group intervention	Standard care	Information-Motivation-Behavioral Skills: ART adherence Depression: Beck Depression Inventory II	No between group difference in adherence motivation and skills, and depression at 3-mo
Robbins (2015)[26]	55 non-adherent South African HIV+ patients on antiretroviral therapy (ART) for at least 6 months Mean age: 38.46y; female: 66.15%; <HS graduate: 98.46%	Masivukeni: an innovative multimedia-based, computer-driven, lay counselor-delivered intervention designed to help people living with HIV in resource-limited settings achieve optimal adherence	Standard care	Social Regulation (medication-specific social support, perceived social support, HIV-related stigma) Self-Regulation (adherence self-efficacy, attitudes towards disclosure, beliefs about medications, HIV/AIDS knowledge)	Int group showed increased medication social support (β = 4.75, *p* = 0.02) and clinic-patient relationships (β = 4.31, *p* = 0.05) and decreased social rejection (β = -2.93, *p* = 0.02) at post-intervention (5–6 weeks after baseline) vs Cont. No between group difference in adherence self-efficacy
Simoni (2007)[21]	136 HIV+ indigent mainly African American and Puerto Rican men and women recruited from an outpatient clinic in the Bronx, New York. Mean age: 42.6y; female: 44.9%; <HS graduate: 43.7%	Peer -led 3-month intervention to address barriers to adherence and sensitively providing appraisal, spiritual, emotional, and informational adherence-related social support	Standard care	Social support: UCLA Social Support Inventory Depressive symptoms: CES-D	No between group difference in social support and depressive symptoms at 6-mo
Van Tam (2012)[24]	A sub-sample study of a randomised controlled trial was implemented between October 2008 and November 2010 in Quang Ninh, Vietnam (N = 228) Int (n = 119): ≤35y: 65.5%; female: 34.5%; HS or higher: 54.6% Cont (n = 109): ≤35y: 73.4%; female: 29.4%; HS or higher: 47.7%	Adherence support from trained peer supporters who visited participants' houses biweekly during the first two months, thereafter weekly	Standard care (adherence counselling, monthly health check and drug refills)	Quality of life: WHOQOL-HIVBREF Internal AIDS-related Stigma (self-blame, concealment of HIV status)	No between group difference in quality of life and internal stigma at 12-mo
Velasquez (2009)[22]	HIV-positive men who have sex with men with alcohol use disorders (N = 253) Mean age: 38.6y; men of color: 79%; ≥HS: 85.4%	Both individual counseling and peer group education/support	Resource referrals	Social support Transtheoretical Model (TTM) constructs	No between group difference in social support and TTM constructs at 12-mo
Webel (2010)[23]	HIV-infected adults who self-identified as female and spoke fluent English (N = 89) Mean age: 47y; female: 73%; <HS graduate: 38.2%; race: black: 76.4%	Seven, peer-led, HIV symptom management using the curriculum, Positive Self-Management Program sessions over seven weeks	A copy of HIV Symptom Management Strategies	Quality of life: HIV/AIDS Targeted Quality of Life Instrument	No between group difference in quality of life at 14-week vs. Cont

Abbreviation: PLWHA, people living with HIV/AIDS; OR, odds ratio; QOL, quality of life

CHW interventions used in the studies varied in focus. For example, 4 studies were focused on promoting prevention andtreatment adherence related self-efficacy [17–19, 26] or self-efficacy for managing chronic disease [20]. Four studies were focused on quality of life [20, 23, 24, 27], 4 on social support [21, 26, 28, 30], and 3 on adherence motivation [19, 25, 26]. Six studies were focused on reducing depressive symptoms [19–21, 25, 27, 28], while 4 studies used CHWs to reduce HIV related stigma [17, 24, 26, 30]. Additional psychosocial outcomes measured in the studies included psychological distress/stress [19], self-advocacy [17], attitudes towards disclosure and beliefs about medication and HIV/AIDS treatment [26], uncertainty in illness [27], readiness for healthful behavior change [28], and hope [29].

### Characteristics, training, and roles of CHWs

Table 2 describes the characteristics, training, and roles of CHWs. All except two [27, 28] articles described the characteristics of CHWs in detail. Of 12 articles that addressed the characteristics of CHWs, 10 reported using at least one peer, defined as an individual living with HIV [17–21, 23, 24, 28–30], while two employed lay health workers trained by a local organization [26] and a HIV case manager, a community leader, and a health care worker [23]. Robbins et al. [26] employed lay counselors trained by a local non-governmental organization, who had previous experience working in clinics performing HIV testing and ART adherence counseling.

**Table 2:** Characteristics, roles, training, and supervision of CHWs.

1^st^ author (Yr)^ref^	Qualification/Characteristics	Roles	Training/Supervision
DeMarco (2013)[17]	Peers met the same inclusion and exclusion criteria as participants; all were Black women over age 40, living with HIV, from similar neighborhoods in Boston. There were 8 groups total; unknown number of leaders.	Intervention leaders led members of groups in writing exercises about their lives following a prompt from a film clip on being a Black woman living with HIV infection. Control group leaders led support groups.	All peer leaders were trained separately in leading groups and in human subjects protections necessary for the project. Intervention leaders learned the Amherst Writers and Artists method of writing following prompts in a supportive environment. Control group leaders were trained to lead a nonspecific support group. The researcher was present in a room next door while groups were conducted; leaders from the intervention and control groups met with graduate students to review the content of sessions. All sessions were digitally recorded.
Enriquez (2014)[28]	One male and one female both living with HIV, who had worked and/or volunteered in HIV care settings, and were chosen for their willingness to participate and with commitment to diversity and language (English and Spanish). Leaders had to receive HIV care at a different clinical site than study participants.	In group sessions, the peer leader helped participants identify barriers to treatment adherence and list ways to overcome them.	Peer leaders were educated about the Ready intervention by researchers in preparation to facilitate groups. To assist in training, researchers played the role of participants for peer leaders.
Fogarty (2001)[18]	Described in Cabral et al. (1996); peer advocates with the HIV intervention group were paraprofessionals with some work experience in health or community programs, and were living with HIV infection.	Peer advocates provided counseling and social support in individual sessions tailored to participants’ needs. These were characterized as “stage of change” encounters targeting specific behaviors, or non-stage of change encounters where advocates assisted participants with social or family needs.	Cabral et al. (1996) recounts the 9-day structured training in stages of change that advocates receive, along with continuing education and review of the method with supervisors.
Gifford (1998)[19]	Two peer leaders (one of whom was HIV-positive) recruited from the local community	Peers led groups in the Positive Self-Management Program, designed to educate participants in self-care behaviors.	4-days of intensive training based on a set protocol; Leaders were provided with a detailed step-by-step manual for conducting the program.
Masquillier (2014)[29]	People living with HIV/AIDS who had been on ART for at least 12 months and who had received a theoretical and practical training on HIV/AIDS, ART and adherence, nutrition and infection control in the home, based on material developed by the researchers.	Peer adherence supporters provided support during individual visits with participants at their home, work or other location, where they assisted with adherence and discussed matters that make adherence more difficult (e.g. stigma), or other issues important to participants. When necessary, supporters referred patients to a clinic.	Peer adherence supporters received theoretical and practical training on HIV/AIDS, ART and adherence, nutrition and infection control in the home, based on material developed by the University of Free State School of Nursing.
Masquillier (2015)[30]	People living with HIV/AIDS who had been on ART for at least 12 months. 98% were female, and the majority had a higher secondary education degree. This article described an intervention also described in Masquillier et al., 2014.	Peer adherence supporters provided help with adherence and discussed any reasons why this could be difficult, such as stigma. They identified possible ART side effects and took action as appropriate. When necessary, the patient was referred to the clinic.	Peer adherence supporters were educated about HIV/AIDS, ART, adherence, infection control at home, and nutrition. The curriculum was based on material developed by the University of the Free State’s School of Nursing. Peer supporters received a monthly stipend of USD $100, conditional on performance.
Molassiotis (2002)[27]	Not described.	Peer-led two-hour groups, where leaders facilitated discussion among group members. In groups, participants were encouraged to describe their feelings about having HIV infection; to identify shared problems, concerns, fears, hopes, and feelings; and to adopt supportive and encouraging roles toward other members of the group.	The same nurse who facilitated the cognitive behavioral therapy intervention also facilitated the peer support/counseling intervention; there was no mention of the peer leaders’ role in the group. The first author also provided training and regular supervision.
Parker (2016)[20]	Peer leader (PL) who spoke English and isiXhosa was identified from the community. PL underwent 40hours of chronic pain management training over 2 week	Peer leader served as an educator by helping participants complete problem solving and goal setting worksheets. PL also led weekly, 2hour aerobic and relaxation classes over a 6week period	Education was provided on the theory and practice of group aerobic exercise. PL received training in goal setting, activity scheduling and the facilitation of group activities. To ensure fidelity of the intervention, sessions led by the PL were video recorded.
Peltzer (2012)[25]	Not described.	Led by a trained lay health worker and adherence counselor, participants received three monthly 1 hour sessions of medication information combined with problem-solving skills in an experiential/interactive group format.	Not described.
Robbins (2015)[26]	Two lay counselors who had received adherence counseling and HIV testing and counseling training and had previous experience working in clinics conducting HIV testing and counseling, as well as ART adherence counseling.	Peer leaders used the Masivukeni multimedia program to assess participants and engage them in a tailored educational and counseling experience over the course of 6 counseling sessions. The program administered standardized screening assessments for psychiatric distress and problems with alcohol and substance use. Scores were automatically and immediately provided to patients, along with scripted messages tailored to patient’s level of impairment, if any.	Counselors were trained in the use of Masivukeni (i.e., how to operate the program and navigate through the intervention) and how to integrate their current adherence counseling skills and knowledge with Masivukeni.
Simoni (2007)[21]	Current clinic patients who were HIV-positive and on HAART served as “peers”. Medical providers identified appropriate candidates.	This was a combination of group leadership and individual relationship-building. There were 6 semi-monthly facilitated meetings between all peer leaders and participants, and each peer leader then followed up with their assigned participants individually. Peer leaders provided navigation assistance, counseling, and social support.	During two separate training sessions over 4 half-days, a total of 12 peers were trained how to assess for negative affective states and other barriers to adherence and to sensitively provide appraisal, spiritual, emotional, and informational social support. They also received training in HIV, HAART, interacting appropriately with peers, and in making referrals for medical and other kinds of care. They were tested at the end of training, and received ongoing supervision through regular phone calls and meetings. Peer leaders were paid a $20–30 incentive depending on the number of participants they worked with.
Van Tam (2012)[24]	Trained people living with HIV who were taking ART.	This was a “peer supporter” role, where the trained peer interacted with participants to inquire about well-being, symptoms, and ART use.	Peer supporters used standardized checklists was developed by the research group together with a group of PLHIV who were on ART to ask questions in a standardized order and manner.
Velasquez (2009)[22]	Nine therapists (master’s- or doctoral-level clinical and counseling psychologists or trainees) and four peer counselors delivered intervention components. The peer counselors who conducted group sessions were self-identifed HIV-positive gay men.	The four peer-led group sessions focused on HIV risk reduction and the adoption and maintenance of safer sexual behaviors. The weekly group session followed the same structure: stage of change assessment/scoring, selection of a process of change-based activity, activity implementation, discussion, and feedback. This approach facilitated group discussion and allowed clients to share their thoughts and experiences with strategies for practicing safer sexual behaviors.	Peer counselors attended a 2-day workshop in conducting brief motivational interventions prior to conducting group sessions. Peer counselors received supervision immediately following each group session and participated in monthly supervision sessions.
Webel (2010)[23]	Three peer leaders were identified as community leaders by HIV case managers, community leaders, and health care workers. There were two trained peer leaders to a group of 10 participants.	The peer leaders served as educators, and followed the Positive Self-Management curriculum for 7 weekly group sessions.	A five-day (total of 36 hours) standardized training on the Positive Self-Management Program curriculum. This scripted training provided structure for leaders and suggestions for coping with problems arising during sessions. Each peer leader led two PSMP modules while the other peer leader and trainers offered constructive feedback on the presentation.

All except one study [25] reported information on CHW training. Studies used didactic methods as well as role-playing to train CHWs to execute designed intervention components. Only DeMarco et al. [17] reported that CHWs received human subject protections training. Peer leaders in Simoni et al. [21] received training in interacting properly with their peers. The length of training ranged from 2 [22] or 4 half-days [21] to 9 days [18]. In one study, CHWs were trained and/or supervised by one of the authors [27]. Only Simoni et al. [21] reported that peers were tested at the end of training and received ongoing supervision via regular phone calls and meetings. Generally, information regarding continuing education during the study period was rarely described. Two studies [21, 30] reported details of payments to CHWs. Simoni et al. [21] paid a $20-$30 incentive depending on the CHW’s assigned number of patients, while Masequillier et al. [30] reported that CHWs received a monthly stipend of $100 depending on their performance.

CHWs played multiple roles in delivering intervention components, resulting in difficulty teasing out particular CHW roles in the study interventions reviewed. In addition to traditional outreach responsibilities of engaging participants in the intervention, CHWs delivered a range of services including education, counseling, social support, and navigation assistance. In most studies [17, 18, 21, 24–30], CHWs fulfilled the role of a counselor and a supporter by helping patients identify barriers to medication adherence. CHWs also discussed potential solutions to problems, and discussed participants’ feelings about having HIV. In some studies, CHWs served as educators [19, 20, 23, 26] or navigators [21, 29, 30]. CHW intervention fidelity and supervision were assessed in 4 studies [17, 19, 20, 27]. In a study by Parker et al. [20], all sessions led by the CHWs were videorecorded to ensure fidelity and that all critical components were addressed.

### Effects of CHW interventions on psychosocial outcomes

Outcomes measured in only one study (e.g., self-advocacy, readiness for healthful behavior change, hope, uncertainty in illness, attitudes towards disclosure) were not synthesized due to a difficulty in comparing findings. Of 14 articles, five reported positive changes in six psychosocial outcomes including self-efficacy in 2 of 4 studies [18, 19], social support in 2 of 4 studies [26, 30], quality of life in 1 of 4 studies [24], and stigma in 1 of 4 studies [26]. Effects of CHW interventions were mixed in promoting self-efficacy. Unlike the other US studies that showed a positive effect of CHWs on self-efficacy [18, 19], two studies conducted in South Africa [20, 26] found non-significance, which is likely associated with a small sample size (n = 27 and 55, respectively).

The effect of a CHW intervention on social support was mixed. Of 4 studies [21, 26, 28, 30], compared to control participants, two studies conducted in South Africa [26, 30] found a significant difference. For example, Robbins et al.[26] found a computer-driven, lay counselor-delivered intervention was effective in promoting medication-specific social support but not in increasing perceived availability of social support among the intervention participants. The remaining two studies [21, 28] had non-significant findings which may have been a result of multiple factors including low attendance in the intervention sessions [21], a small sample size (n = 20) [28], or the insufficient power to detect differences in social support as a secondary outcome of the study (i.e., medication adherence was main outcome) [28].

Of 4 studies that assessed quality of life, only one [24] implemented in Vietnamese outpatient clinics reported a significant difference in quality of life after 12-months in the intervention participants. This study included biweekly counseling for the first two months and weekly thereafter. In contrast, the remaining three studies [20, 23, 27] found no improvement in quality of life. For example, in a study to test peer-led, six-week sessions on physical exercise and problem solving education to promote chronic pain management among 27 women living with HIV recruited from a community health center in South Africa [20], Parker et al. found no improvement in quality of life which might have been a result from the small sample size used for the experimental (n = 12) and control group (n = 15). Similarly, Webel et al. [23] tested peer-led, seven-week sessions on HIV symptom management and found positive changes in two of the nine quality of life scales only, including HIV mastery (chi square = 25.08; p<0.005) and disclosure worries (chi square = 24.67; p<0.005) among the intervention participants, but the effect became insignificant after controlling for baseline data. Molassiotis et al.[27] assessed the effect of cognitive behavioral therapy (n = 10; 2 hrs of weekly sessions for 12 weeks) and peer support/counseling group therapy (n = 10) compared to a group receiving standard care (n = 26) in a Hong Kong outpatient clinic. Although quality of life scores improved over time in the cognitive behavioral theory group (p<0.001; 6.7% improvement), no intervention effect was noted in the cognitive behavioral therapy and the peer support/counseling group therapy groups compared to the standard care group.

Stigma was measured as an outcome in 4 studies [17, 24, 26, 30] and one study found a significant reduction in stigma/social rejection after a lay counselor-delivered tailored education and counseling intervention [26]. Possible explanations for the non-signficant findings may be methodological or conceptual in nature. For example, DeMarco et al. [17] stated a short-term intervention (i.e., 4 weeks) as an explanation for the non-significant finding. In some cultural context (e.g., Vietnamese), the use of CHWs may not be effective in decreasing social stigma (e. g., problem getting a job, unfair treatment in the workplace or healthcare setting) [24]. Masquillier et al. [30] speculated that emphasizing anonymity and confidentiality in the study might actually have produced adverse intervention effects that is, increased levels of stigma.

All studies measuring depressive symptoms [19–21, 25, 27, 28] found non-significant difference between groups. Simoni et al. [21] noted that the non-significant result might have had to do with insufficient exposure of the intervention participants to the intervention because only 17% of them attended five or more out of 6 sessions offered. In addition to small sample sizes used in the studies [19, 20, 27, 28], the non-significant findings might have been associated with unbalanced allocation to the intervention or the control groups as noted in 3 studies [19, 20, 27].

## Discussion

Systematic reviews examining CHW interventions for psychosocial outcomes in PLWH have been scarce. The studies included in the systematic review provide some evidence for the use of CHWs in promoting psychosocial outcomes in PLWH. Even though interventions utilizing CHWs within social networks was among the most frequently employed intervention approaches [2], CHW interventions were associated with small or inconsistent efficacy in addressing psychosocial outcomes in PLWH.

While depressive symptoms, self-efficacy, quality of life, social support, and stigma were among the most popular psychosocial outcomes measured, CHW interventions were proven effective only in some of the studies. In particular, none of the CHW interventions included in the review were successful in achieving a statistically significant reduction in depressive symptoms among PLWH in intervention groups in comparison to those in control groups. Depression symptomatology was varied in these studies and none described the use of anti-depressants for control of such symptoms. It remains unclear the impact of CHW interventions in setting where treatment of depressive symptoms with peer support is available. The findings may have been due, in part, to methodological biases associated with the studies included in the review: small sample size [19, 26–28], use of unstandardized psychosocial outcome measures (i.e., outcome measure developed for the purpose of the study) [18, 19], high attrition rates [17, 18, 27, 29, 30], short follow-up [19–21, 23, 25, 26], or lack of a priori power analysis [18, 24, 29, 30]. Most often, available a priori sample size calculation was focused on addressing clinical outcomes (e.g., viral load) as a primary outcome [20, 21, 23, 25]; psychosocial outcomes were addressed as secondary outcomes and the studies rarely described any power analysis to focus on the psychosocial variables.

Training, leading to competency development may be another possible explanation for the lack or inconsistent efficacy of CHW interventions. While the majority of the studies included in the review described the characteristics, qualification, and training of CHWs, very limited information was provided in terms of how CHW competency was verified at the end of training [21]. Furthermore, CHW intervention fidelity and supervision were assessed in less than one third of the studies included [17, 19, 20, 27]. Inadequate descriptions of the CHW competency verification and monitoring in the interventions make it difficult to explain why this approach was not effective. Without sufficient monitoring, the utility of CHW intervention is unclear because the content and frequency of interactions between the CHW and the participant can vary widely, at the discretion of the CHW [31]. The lack of significant positive effect in the studies may suggest the need for more rigorous CHW training and monitoring plans to improve the efficacy of CHW interventions focusing on psychosocial outcomes among PLWH. In particular, CHW competency measure along with more systematic methods to assess and confirm CHW fidelity would be warranted.

### Review limitations

The strengths of this review’s design included its inclusive search strategy that ensured wide coverage, standardized data extraction, and iterative analysis. A limitation of our analysis is the heterogeneity in the quality and quantity of data reported in the 14 studies. In an attempt to address this issue, we have provided quality ratings for each study included in the review. In addition, we were unable to conduct more exhaustive searches of the gray literature and did not include studies in languages other than English, hence limiting the generalizability of our findings. Lastly, we only focused on CHW interventions using an RCT design; the findings of this review cannot be generalized to other studies of CHWs using non-RCT method (e.g., quasi-experimental).

### Research agenda

This review has revealed several gaps in the existing evidence base, gaps that collectively point to what we argue should be key parts of the research agenda going forward. The most important gap, and a critical focus of future research, is the inclusion of concepts from the social determinants of health which do not appear in these studies. Much of the research we reviewed was focused on the same variables but with little impact. Specifically, CHW interventions were focused on reducing depression (n = 6) followed by promoting prevention/treatment adherence self-efficacy (n = 4) and increasing quality of life or social support (n = 4 each) while reducing stigma related to HIV (n = 4). Future CHW intervention should be expanded in scope to address key psychosocial determinants of HIV/AIDS outcomes such as health literacy [31]. There is also limited explanation of psychosocial variables chosen in the literature. Selection of study outcomes was minimally justified within the reviewed studies. This underscores the need for adopting an integrative psychoecological framework to better understand health behaviors and outcomes of PLWH. Finally, this review highlights a critical methodological gap and area for future improvement–the need for ensuring sufficient power with adequate sample size, use of validated psychosocial outcome measures, and longer-term follow up of study variables. Further, CHW fidelity measures should be incorporated into intervention delivery as standard procedures. This review suggests that CHWs intervention effects on psychosocial outcomes would benefit greatly from research that used consistent, standardized, and appropriate measures of outcomes.

### Conclusions

The results of this systematic review indicate that CHW interventions may have limited efficacy in addressing psychosocial outcomes. At this point, it is unclear whether a CHW strategy can significantly improve these psychosocial outcomes among PLWH, given the inconsistent evidence in support of this approach. While interventions based on social networks, such as the use of CHWs, have been popular, the outcomes obtained from this approach were inconsistent and usually not positive. Future research using this approach should include rigorous training of CHWs using competence evaluation and a well-designed monitoring plan. Finally, additional, tightly controlled randomized trials are needed to improve the efficacy of CHWs on involving PLWH.

## Appendix 1. Search strategies

### PubMed

((((((hiv infections[mh] OR hiv[mh] OR hiv[tw] OR hiv-1[tw] OR hiv-2[tw] OR hiv1[tw] OR hiv2[tw] OR hiv infect*[tw] OR human immunodeficiency virus[tw] OR human immunedeficiency virus[tw] OR human immuno-deficiency virus[tw] OR human immune-deficiency virus[tw] OR ((human immun*) AND (deficiency virus[tw])) OR acquired immunodeficiency syndrome[tw] OR acquired immunedeficiency syndrome[tw] OR acquired immuno-deficiency syndrome[tw] OR acquired immune-deficiency syndrome[tw] OR ((acquired immun*) AND (deficiency syndrome[tw])))) AND (community health workers [mh] OR "community health worker" OR "community health workers" OR "lay health worker" OR "lay health workers" OR "lay health advisor" OR "lay health advisors" OR "lay health counselors" OR "chw" OR "lhw" OR "lha" OR "lhc" OR peer [tiab] OR peers [tiab]))))) AND interven* [tw]

### CINAHL

((MH "HIV Infections+") OR "hiv" OR (MH "Human Immunodeficiency Virus+") OR hiv*) OR (human immunodeficiency virus OR humanimmunedeficiency virus oR human immuno-deficiency virus OR human immune-deficiency virus OR (human immun*) N3 (deficiency virus)) OR (acquired immunodeficiency syndrome OR acquired immune-deficiency syndrome OR acquired immunedeficiency syndrome OR acquired immuno-deficiency syndrome OR (acquired immune) N3 (deficiency syndrome)) AND ((MM "Community Health Workers") OR "community health worker" OR "community health workers" OR "lay health worker" OR "lay health workers" OR "lay health advisor" OR "lay health advisors" OR "lay health counselor" OR "lay health counselors" OR "chw" OR "lhw" OR "lha" OR "lhc" OR peer OR peers)) AND interven*

### Embase

(('health auxiliary'/exp OR 'health auxiliary' OR 'community health worker':ab,ti OR 'community health workers':ab,ti OR 'lay health worker':ab,ti OR 'lay health workers':ab,ti OR 'lay health advisor' OR 'lay health advisors' OR 'lay health counselor' OR 'lay health counselors' OR chw:ab,ti OR lhw:ab,ti OR lha:ab,ti OR lhc:ab,ti OR peer:ab,ti OR peers:ab,ti) AND ('human immunodeficiency virus infection'/exp OR 'human immunodeficiency virus'/exp OR 'acquired immune deficiency syndrome'/exp OR 'hiv':ab,ti OR 'hiv-1':ab,ti OR 'hiv-2':ab,ti OR 'hiv1':ab,ti OR 'hiv2':ab,ti OR 'human immunodeficiency virus':ab,ti OR 'human immunedeficiency virus':ab,ti OR 'human immuno-deficiency virus':ab,ti OR 'human immune-deficiency virus':ab,ti OR (human NEAR/4 immune* AND deficiency NEAR/4 virus) OR 'acquired immunodeficiency syndrome':ab,ti OR 'acquired immunedeficiency syndrome':ab,ti OR 'acquired immuno-deficiency syndrome':ab,ti OR 'acquired immune-deficiency syndrome' OR (acquired NEAR/4 immun* AND deficiency NEAR/4 syndrome))) AND (interven*:ab,ti OR intervention)

### Cochrane

#1 MeSH descriptor: [Community Health Workers] explode all trees

#2 community health worker or community health workers or lay health worker or lay health workers or lay health advisor or lay health advisors or lay health counselor or lay health counselors or chw or lhw or lha or lhc or peer or peers

#3 #1 or #2

#4 MeSH descriptor: [HIV Infections] explode all trees

#5 MeSH descriptor: [HIV] explode all trees

#6 hiv* or hiv near/3 infect* or ((human or acquir*) near/3 immune* near/3 (virus or syndrome))

#7 #4 or #5 or #6

#8 interven*

#9 #3 and #7 and #8

## Supporting information

S1 PRISMA Checklist (DOC)
